# Antenatal oral glucose tolerance test abnormalities in the prediction of future risk of postpartum diabetes in women with gestational diabetes: Results from the LIVING study

**DOI:** 10.1111/1753-0407.13559

**Published:** 2024-05-06

**Authors:** Yashdeep Gupta, Deksha Kapoor, Josyula K. Lakshmi, Devarsetty Praveen, Joseph Alvin Santos, Laurent Billot, Aliya Naheed, H. Asita de Silva, Ishita Gupta, Noshin Farzana, Renu John, Saumiyah Ajanthan, Neerja Bhatla, Ankush Desai, Arunasalam Pathmeswaran, Dorairaj Prabhakaran, Helena Teede, Sophia Zoungas, Anushka Patel, Nikhil Tandon

**Affiliations:** ^1^ Department of Endocrinology and Metabolism All India Institute of Medical Sciences New Delhi India; ^2^ George Institute for Global Health Hyderabad India; ^3^ Faculty of Medicine and Health University of New South Wales Sydney New South Wales Australia; ^4^ Prasanna School of Public Health Manipal Academy of Higher Education Manipal India; ^5^ George Institute for Global Health Sydney New South Wales Australia; ^6^ Initiative for Non Communicable Diseases, Nutrition Research Division International Centre for Diarrhoeal Disease Research (ICDDR, B) Dhaka Bangladesh; ^7^ Clinical Trials Unit, Department of Pharmacology, Faculty of Medicine University of Kelaniya Kelaniya Sri Lanka; ^8^ Centre for Chronic Disease Control New Delhi India; ^9^ RemediumOne Colombo Sri Lanka; ^10^ Department of Obstetrics and Gynaecology All India Institute of Medical Sciences New Delhi India; ^11^ Department of Endocrinology Goa Medical College Goa India; ^12^ Department of Public Health, Faculty of Medicine University of Kelaniya Ragama Sri Lanka; ^13^ Public Health Foundation of India New Delhi India; ^14^ London School of Hygiene and Tropical Medicine London UK; ^15^ Monash Centre for Health Research and Implementation, School of Public Health and Preventive Medicine Monash University Melbourne Victoria Australia; ^16^ School of Public Health and Preventive Medicine Monash University Melbourne Victoria Australia

**Keywords:** antenatal OGTT, gestational diabetes mellitus, postpartum, risk prediction, South Asia, type 2 diabetes mellitus

## Abstract

**Objectives:**

To explore associations between type and number of abnormal glucose values on antenatal oral glucose tolerance test (OGTT) with postpartum diabetes in South Asian women diagnosed with gestational diabetes (GDM) using International Association of the Diabetes and Pregnancy Study Groups criteria.

**Methods:**

This post‐hoc evaluation of the Lifestyle Intervention IN Gestational Diabetes (LIVING) study, a randomized controlled trial, was conducted among women with GDM in the index pregnancy, across 19 centers in Bangladesh, India, and Sri Lanka. Postpartum diabetes (outcome) was defined on OGTT, using American Diabetes Association (ADA) criteria.

**Results:**

We report data on 1468 women with GDM, aged 30.9 (5.0) years, and with median (interquartile range) follow‐up period of 1.8 (1.4–2.4) years after childbirth following the index pregnancy. We found diabetes in 213 (14.5%) women with an incidence of 8.7 (7.6–10.0)/100 women‐years. The lowest incidence rate was 3.8/100 women years, in those with an isolated fasting plasma glucose (FPG) abnormality, and highest was 19.0/100 women years in participants with three abnormal values. The adjusted hazard ratios for two and three abnormal values compared to one abnormal value were 1.73 (95% confidence interval [CI], 1.18–2.54; *p* = .005) and 3.56 (95% CI, 2.46–5.16; *p* < .001) respectively. The adjusted hazard ratio for the combined (combination of fasting and postglucose load) abnormalities was 2.61 (95% CI, 1.70–4.00; *p* < .001), compared to isolated abnormal FPG.

**Conclusions:**

Risk of diabetes varied significantly depending upon the type and number of abnormal values on antenatal OGTT. These data may inform future precision medicine approaches such as risk prediction models in identifying women at higher risk and may guide future targeted interventions.

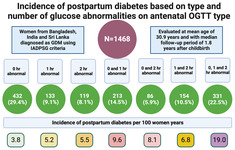

## Graphical abstract

Highlights

- Of the 1468 South Asian women with gestational diabetes, 213 (14.5%) women developed diabetes over median follow‐up 1.8 years after childbirth, an incidence rate of 8.7/100 women‐years.
- The incidence rates for participants with any one, two, and three abnormal values on antenatal oral glucose tolerance test (OGTT) were 4.3, 8.3 and 19.0/100 women years.
- The rates of future diabetes in this high‐risk cohort varied considerably with antenatal OGTT values, providing the opportunity for targeted intervention to prevent future diabetes.

## 1 INTRODUCTION

Gestational diabetes mellitus (GDM) is a risk factor for future diabetes and cardiovascular diseases.^1^ Guidelines advise that women with GDM must be followed annually after childbirth, evaluated preferably with an oral glucose tolerance test (OGTT), and by less labor‐intensive tests like fasting plasma glucose (FPG) or glycated hemoglobin (HbA1c), where OGTT is not feasible. If a woman is found to have prediabetes/diabetes, she should be offered lifestyle modification with or without pharmacotherapy.^2^, ^3^ The annual burden of GDM globally is estimated at 16.9 million.^1^, ^4^ Widespread postpartum screening for diabetes has resource implications. It presents a significant burden on individuals and the healthcare system, especially in low and middle‐income countries (LMICs), which are often poorly equipped to handle this load.^5^, ^6^ Therefore, the concept of precision medicine is gaining prominence among models of post‐GDM care.^7^ Identifying high‐risk patients helps concentrate resources for individuals at immediate and highest risk of developing disease. Pragmatic implementation requires risk identification using simple, routinely available health data.

Previous research has examined the association between routinely available antenatal OGTT values and future incident diabetes. The type of abnormality (fasting or postglucose load), the number of abnormal values, and the various cutoff points have been explored. Moore et al investigated associations between fasting and 2‐h post OGTT glucose values with future diabetes in 17 studies.^7^ Notably, none of the included studies in this analysis used the International Association of the Diabetes and Pregnancy Study Groups (IADPSG) criteria, which are currently widely adopted. Further, the meta‐analysis was not possible for 1‐h values due to inadequate data. Two recent additional studies, from Canada (*n* = 20 513 with GDM; median follow‐up of 4.4 years) and Singapore (*N* = 942 with GDM, evaluated at 6–12 weeks postpartum), explored the relationship between the OGTT type and the future risk of diabetes for women diagnosed using IADPSG criteria.^8^, ^9^ Both studies found significant differences in future risk of diabetes based on OGTT type. Given that GDM diagnosed using IADPSG criteria is milder with a lower risk of future diabetes than that with other criteria, identifying high‐risk women with a history of IADPSG criteria becomes even more crucial for judicious use of limited resources.^10^, ^11^, ^12^

Given the high risk of developing diabetes after GDM in South Asia and the limited data in LMICs using IADPSG criteria, we aimed to complete a Lifestyle Intervention IN Gestational Diabetes (LIVING) trial substudy to explore associations between type and number of abnormal glucose values on antenatal OGTT with postpartum diabetes in South Asian women diagnosed with past GDM using IADPSG criteria.

We seek to address key gaps and enhance understanding of diabetes risks to inform prevention approaches considering limited resources in LMICs.

## 2 METHODS

### 2.1 Settings and study design

This study is a post‐hoc analysis of the LIVING trial.^13^ This study represents a prospective cohort study within a randomized trial. Because there were no differences among women for incident diabetes in both randomized arms, data from both arms were combined. In addition, we included women who were not randomized (either due to diabetes or other reasons) but were part of the prerandomization assessment (details provided ahead). LIVING was an investigator‐initiated trial involving 19 clinical centers in Bangladesh, India, and Sri Lanka. Ethics Review Committees of the All India Institute of Medical Sciences (India), Centre for Chronic Disease Control (India), ICDDR,B (Bangladesh), Faculty of Medicine, University of Kelaniya (Sri Lanka), the University of Sydney (Australia), and (where required) individual hospitals approved the study. We obtained written informed consent from all study participants.

### 2.2 Trial overview

In the LIVING trial,^13^ we evaluated women with a recent diagnosis of GDM. We randomized 1601 women who did not have diabetes at the first postpartum visit (prerandomization) in 1:1 to lifestyle intervention vs usual care. The primary outcome was worsening of glycemia (normal to prediabetes/diabetes or prediabetes to diabetes) based on OGTT using American Diabetes Association criteria.^14^ The intervention did not affect the primary outcome with no difference in glycemic status compared to usual care (25.5% vs 27.1%; hazard ratio, 0.92 [95% confidence interval (CI), 0.76–1.12]; *p* = .42). Due to the lack of significant differences in the primary and secondary outcomes, including weight, and diabetes rates, we used the dataset from both arms for the current study. In addition, we included those who underwent testing (postpartum OGTT) as part of the baseline visit but did not undergo randomization either due to the detection of diabetes or were excluded/not enrolled in the trial for other reasons.

### 2.3 Participant numbers for this study

**FIGURE 1:**
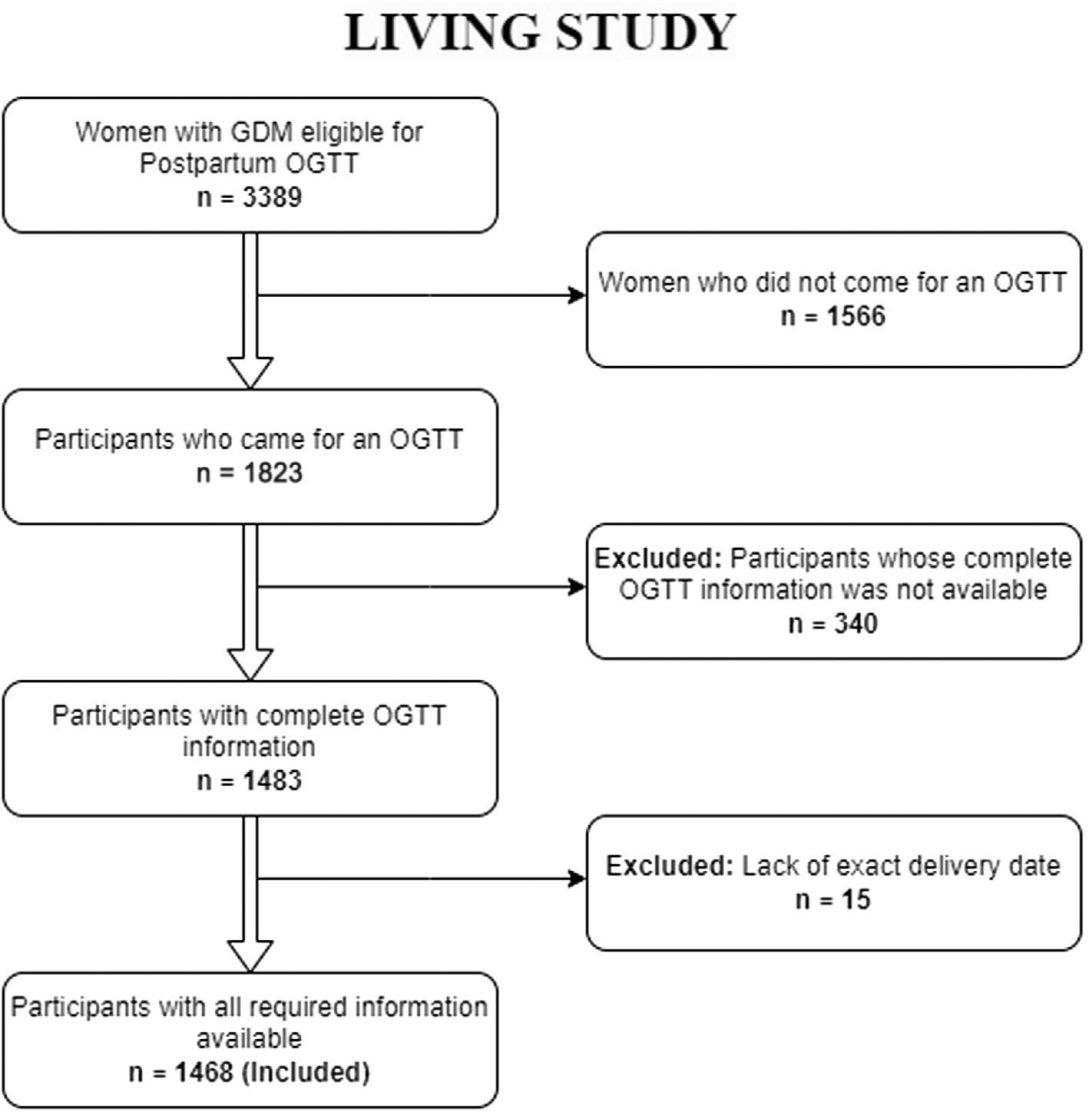
Study flow of this LIVING substudy. GDM, gestational diabetes; LIVING, Lifestyle Intervention IN Gestational Diabetes; OGTT, oral glucose tolerance test.

In the LIVING study, out of 3389 registered participants with GDM, 1823 individuals had an OGTT at the first postpartum visit. Of these, 160 individuals (8.8%) had type 2 diabetes, 51 individuals were excluded for other reasons (2 women [0.1%] met other exclusion criteria, 49 women [2.7%] did not consent or were uncontactable), and 1612 individuals were randomized. Eleven randomized participants were subsequently identified as ineligible and excluded from the primary analysis, leaving 1601 women.^5^ This substudy included all these 1823 individuals except 340, for whom we did not have information on all three values of antenatal OGTT or were diagnosed before 24 weeks gestation and 15, for whom we lacked information on the date of delivery required for this analysis. The final sample size for this study is 1468 (Figure 1). Overall, this analysis represents a longitudinal cohort within a randomized controlled trial. When the event of interest (diabetes) was captured at prerandomization visits, such participants were excluded from the trial. Data from these participants would similarly also have been censored if the primary investigative approach had been a predesigned prospective cohort study to assess factors causally associated with incident diabetes. All other participants without diabetes were included in the randomized controlled trial and further followed up for incident diabetes (the same as would have been done if it been a predesigned prospective cohort study). As the first assessment visit for this study happened after the standard 6–12 weeks window, it is challenging to classify diabetes found at prerandomization visits as prevalent (preexisting) or incident diabetes. Some cases may represent the former. Further planned termination of follow‐up occurred for the randomized participants if a participant became pregnant.

### 2.4 Study objectives

This study aimed to assess the association between type of abnormalities in glucose values on antenatal OGTT and the incidence of diabetes after childbirth (outcome variable) in women with GDM in an index pregnancy using three glucose‐based classifications.

### 2.5 Classifications based on exposure variable

We explored and compared the incidence of diabetes across these three different classifications.

1. For (a) isolated abnormal 0 h value (FPG) (1‐ and 2‐h values being normal), (b) isolated abnormal 1 h value (0 and 2‐h values being normal), (c) isolated abnormal 2‐h value (0 and 1‐h values being normal), (d) abnormal 0 and 1‐h values (2‐h value being normal), (e) abnormal 0 and 2‐h values (1‐h value being normal), (f) abnormal 1‐ and 2‐h values (0 h value being abnormal), and (g) all abnormal values (0, 1‐, and 2‐h values).

2. Isolated abnormal FPG (only 0 h value abnormal), isolated postglucose load abnormality (1‐ and/or 2‐h value(s) abnormal) and combination of fasting (0 h) and postglucose load (1‐ and/or 2‐h) abnormalities.

3. For one (isolated 0, or 1‐ or 2‐h value, categories a, b, or c as defined under point 1), two (categories d, e, or f as defined under point 1) or three (category g as defined under point 1) abnormal values on OGTT.

The abnormal values for 0, 1‐, and 2‐h were defined as per the IADPSG criteria, that is, 0 h ≥92 mg/dL (≥5.2 mmol/L), 1 h ≥180 mg/dL (≥ 10 mmol/L), and 2 h ≥153 mg/dL (≥8.5 mmol/L).^15^

### 2.6 Participant identification, inclusion and exclusion criteria

Eligible study participants were women diagnosed with GDM using IADPSG criteria based on OGTT results at 24–34 weeks of gestation, except for those excluded due to lack of complete information. Trial exclusion criteria also included;

1. Travel time to hospital >2 h,

2. Lack of availability of a household mobile telephone,

3. Use of steroids during pregnancy (other than for fetal lung maturation), and

4. High likelihood of moving residence within the subsequent 3 years.

### 2.7 Procedure on the day of testing

We invited the participants for a baseline visit and OGTT (minimum fast of 8 hours) between 3 and 18 months following childbirth. We obtained venous plasma glucose in the fasting state, and 2 h after ingestion of 82.5 g of glucose monohydrate (equivalent to 75 g of anhydrous glucose), dissolved in 250–300 mL water and consumed over 5–10 min. We collected information on demographics, education, employment, prior history of GDM (before the index pregnancy), and other relevant medical/obstetric history at the baseline visit.

### 2.8 Definitions of outcomes

Postpartum glycemic and diabetes categories were defined based on fasting [≥7.0 mmol/L] and 2‐h [≥11.1 mmol/L] blood glucose levels from the OGTT.^14^ The outcome was measured at the first postpartum visit (the prerandomization visit conducted 3–18 months after delivery) for the women who were not randomized and at the last available follow‐up for the randomized participants. The follow‐up was conducted every 6 months, to a maximum of 36 months from randomization visit for the randomized participants.

### 2.9 Statistical analysis

We have presented data using descriptive statistics, as frequency and proportion for categorical variables and as mean and SD or median and interquartile range for continuous variables. Person‐time and incidence rate of diabetes (ie, the number of events divided by the person time) were calculated from the date of childbirth to the diagnosis of diabetes (for participants who experienced the event, ie, developed diabetes) or to the last available OGTT (for participants who did not experience the event, ie, did not develop diabetes). Median follow‐up time was calculated by reversing the Kaplan–Meier estimate as suggested by Schemper and Smith.^16^ Cox proportional hazards model was used to analyze the time to the development of diabetes with the study center as a random effect. Adjusted analyses included age, education, employment, prior GDM history, family history of diabetes, pregnancy count, and body mass index (BMI) category. The covariates selected for adjustment are well‐known risk factors for diabetes and can have an independent association with diabetes. Analyses were conducted in STATA BE V17.0 for Windows (StataCorp LLC, College Station, TX, USA). No imputation for missing data was conducted.

## 3 RESULTS

### 3.1 Baseline characteristics

We evaluated 1468 women with mean age (SD) of 30.9 (5.0) years. The median follow‐up after childbirth following the index pregnancy was 1.8 (1.4–2.4) years. There were 503 (35.3%) women with education higher than secondary school, and 241 (16.9%) were employed. Prior history of GDM (other than in the index pregnancy) and family history of diabetes were present in 96 (6.7%) and 648 (45.5%), respectively. There were 298 (21.0%) women who were obese (BMI ≥30 kg/m^2^), 548 (38.7%) who were overweight (BMI: 25.0–29.9 kg/m^2^), and 42 (3.0%) who were underweight (BMI <18.5 kg/m^2^). We found diabetes in 213 (14.5%) women with an incidence rate of 8.7 (7.6–10.0)/100 women‐years. The baseline characteristics are presented in Table 1.

**TABLE 1:** Baseline characteristics.

Variables	Total *N* = 1468
Age, (years) (mean, SD)	30.9 (5.0)
Religion (*n*, %)	
Buddhist	262 (18.4%)
Christian	150 (10.5%)
Hindu	564 (39.6%)
Muslim	416 (29.2%)
Sikh	28 (2.0%)
Other	4 (0.3%)
Education (*n*, %)	
Secondary school or below	920 (64.7%)
Higher than secondary school	503 (35.3%)
Employment (*n*, %)	
Unemployed	1182 (83.1%)
Employed	241 (16.9%)
Gravida (median, IQR)	2.0 (1.0–3.0)
Prior history of gestational diabetes (*n*, %)	
No	1327 (93.3%)
Yes	96 (6.7%)
Family history of diabetes in first‐degree relatives (*n*, %)	
No	775 (54.5%)
Yes	648 (45.5%)
Body weight, kg (mean, SD)	63.0 (11.7)
Body mass index, kg/m^2^ (mean, SD)	26.5 (4.6)
Body mass index classification (*n*, %)	
Underweight	42 (3.0%)
Normal weight	529 (37.3%)
Overweight	548 (38.7%)
Obese	298 (21.0%)
Waist circumference, cm (mean, SD)	89.2 (11.9)
Systolic blood pressure, mmHg (mean, SD)	112.9 (11.4)
Diastolic blood pressure, mmHg (mean, SD)	74.8 (9.1)
Fasting plasma glucose during pregnancy, mg/dL (mean, SD)	96.4 (10.8)
Glucose 1 h post OGTT during pregnancy, mg/dL (mean, SD)	179.7 (31.0)
Glucose 2 h post OGTT during pregnancy, mg/dL (mean, SD)	146.8 (29.0)

*Note*: N varies by variable. BMI classification: underweight (<18.5 kg/m^2^); normal weight (18.5–24.9 kg/m^2^); overweight (25.0–29.9 kg/m^2^); obese (>30.0 kg/m^2^).

Abbreviations: BMI, body mass index; IQR, interquartile range; OGTT, oral glucose tolerance test.

### 3.2 Association of abnormal values on OGTT either isolated or in various combinations with future diabetes

Elevated 0 h (FPG), 1‐h, and 2‐h values on antenatal OGTT were present in 72.3%, 56.6%, and 47.0% of participants (Table 2). The incidence rates for future diabetes based on 0 h, 1‐h, and 2‐h values in the study were 9.8, 11.9, and 12.4/100 women‐years, respectively (Table 3). There were 46.6% of the participants who had only one value elevated on antenatal OGTT, and 22.5% had all elevated values. Of the participants with only one elevated value, 29.4%, 9.1%, and 8.1% had elevated values at 0 h, 1 h, and 2 h, respectively. In participants with dual elevated values, 0 and 1‐h values were the most common combination (Table 2). The incidence rate of diabetes was 19.0/100 women years (15.6 to 23.1) in those with all elevated values compared to 3.8/100 women years in those with only isolated FPG abnormality (Table 3), with an adjusted hazard ratio of 3.60 (95% CI, 2.30–5.64; *p* < .001). The adjusted hazard ratio was also significantly elevated ((1.85 [95% CI, 1.10–3.11; *p* = .020] for the 0 and 1‐h combination compared to the isolated FPG elevation (Table 4).

**TABLE 2:** Distribution of participants based on different classifications of abnormal antenatal oral glucose tolerance test.

Variables	Total *N* = 1468
FPG ≥ 92 mg/dL (*n*, %)	
No	406 (27.7%)
Yes	1062 (72.3%)
Glucose 1‐h post OGTT ≥ 180 mg/dL (nn, %)	
No	637 (43.4%)
Yes	831 (56.6%)
Glucose 2‐h post OGTT ≥ 153 mg/dL (*n*, %)	
No	778 (53.0%)
Yes	690 (47.0%)
Classification 1 (*n*, %)	
Only FPG abnormal	432 (29.4%)
Only 1‐h post OGTT abnormal	133 (9.1%)
Only 2‐h post OGTT abnormal	119 (8.1%)
Abnormal FPG and 1‐h post OGTT	213 (14.5%)
Abnormal FPG and 2‐h post OGTT	86 (5.9%)
Abnormal 1‐h and 2‐h post OGTT	154 (10.5%)
All measures abnormal	331 (22.5%)
Classification 2 (*n*, %)	
Only FPG abnormal	432 (29.4%)
Normal FPG and abnormal 1‐h or 2‐h post OGTT	406 (27.7%)
Abnormal FPG and abnormal 1‐h or 2‐h post OGTT	630 (42.9%)
Classification 3 (*n*, %)	
1 of 3 deranged	684 (46.6%)
2 of 3 deranged	453 (30.9%)
3 of 3 deranged	331 (22.5%)

Abbreviations: FPG: fasting plasma glucose during pregnancy; 1‐h post OGTT: glucose 1‐hour post OGTT during pregnancy; 2‐h post OGTT: glucose 2‐h post OGTT during pregnancy.

**TABLE 3:** Incidence rates of diabetes per 100 person‐years based on different classifications of abnormal antenatal oral glucose tolerance test.

Classification	Person‐time	Events	Incidence rate (95% CI)
Overall	2443.8	213	8.7 (7.6–10.0)
Classification 1			
Only FPG abnormal	742.9	28	3.8 (2.6–5.5)
Only 1‐h post OGTT abnormal	229.3	12	5.2 (3.0–9.2)
Only 2‐h post OGTT abnormal	200.8	11	5.5 (3.0–9.9)
Abnormal FPG and 1‐h post OGTT	345.3	33	9.6 (6.8–13.4)
Abnormal FPG and 2‐h post OGTT	135.3	11	8.1 (4.5–14.7)
Abnormal 1‐h and 2‐h post OGTT	264.5	18	6.8 (4.3–10.8)
All measures abnormal	525.8	100	19.0 (15.6–23.1)
Classification 2			
Only FPG abnormal	742.9	28	3.8 (2.6–5.5)
Normal FPG and abnormal 1‐h or 2‐h post OGTT	694.6	41	5.9 (4.3–8.0)
Abnormal FPG and abnormal 1‐h or 2‐h post OGTT	1006.3	144	14.3 (12.2–16.8)
Classification 3			
1 of 3 deranged	1173.0	51	4.3 (3.3–5.7)
2 of 3 deranged	745.0	62	8.3 (6.5–10.7)
3 of 3 deranged	525.8	100	19.0 (15.6–23.1)
FPG ≥ 92 mg/dL			
No	694.6	41	5.9 (4.3–8.0)
Yes	1749.2	172	9.8 (8.5–11.4)
Glucose 1‐h post OGTT ≥ 180 mg/dL			
No	1079.0	50	4.6 (3.5–6.1)
Yes	1364.8	163	11.9 (10.2–13.9)
Glucose 2‐h post OGTT ≥ 153 mg/dL			
No	1317.5	73	5.5 (4.4–7.0)
Yes	1126.3	140	12.4 (10.5–14.7)

*Note*: The median follow‐up time is 1.8 (IQR 1.4 to 2.4) years. The total number of events is 213 of 1468 (14.5%).

Abbreviations: CI, confidence interval; FPG: fasting plasma glucose during pregnancy; 1‐h post OGTT: glucose 1‐hour post OGTT during pregnancy; 2‐h post OGTT: glucose 2‐h post OGTT during pregnancy.

**TABLE 4:** Hazard ratios based on different classifications of abnormal antenatal oral glucose tolerance test.

Variables	Unadjusted		Adjusted	
	HR (95% CI)	*p* value	HR (95% CI)	*p* value
Classification 1				
Only FPG abnormal	Ref	Ref	Ref	Ref
Only 1‐h post OGTT abnormal	1.35 (0.69– 2.66)	.384	1.05 (0.53–2.07)	.898
Only 2‐h post OGTT abnormal	1.29 (0.63–2.67)	.484	1.00 (0.47–2.14)	.998
Abnormal FPG and 1‐h post OGTT	2.65 (1.60–4.38)	<.001	1.85 (1.10–3.11)	.020
Abnormal FPG and 2‐h post OGTT	2.22 (1.11–4.47)	.025	1.64 (0.79–3.43)	.186
Abnormal 1‐h and 2‐h post OGTT	1.81 (1.00–3.28)	.049	1.65 (0.90–3.02)	.107
All measures abnormal	5.04 (3.31–7.66)	<.001	3.60 (2.30–5.64)	<.001
Classification 2				
Only FPG abnormal	Ref	Ref	Ref	Ref
Normal FPG and abnormal 1‐h or 2‐h post OGTT	1.51 (0.93–2.44)	.097	1.20 (0.73–1.97)	.471
Abnormal FPG and abnormal 1‐h or 2‐h post OGTT	3.86 (2.58–5.79)	<.001	2.61 (1.70–4.00)	<.001
Classification 3				
1 of 3 deranged	Ref	Ref	Ref	Ref
2 of 3 deranged	2.02 (1.39–2.94)	<.001	1.73 (1.18–2.54)	.005
3 of 3 deranged	4.49 (3.20–6.31)	<.001	3.56 (2.46–5.16)	<.001

*Note*: Cox‐proportional hazards model, with study center as random effect. Adjusted for age, education, employment, gravida, history of gestational diabetes mellitus, family history of diabetes, and body mass index classification.

Abbreviations: CI, confidence interval; FPG: fasting plasma glucose during pregnancy; HR, hazard ratio; 1‐h post OGTT: glucose 1‐hour post OGTT during pregnancy; 2‐h post OGTT: glucose 2‐h post OGTT during pregnancy.

### 3.3 Association of isolated fasting, isolated postglucose load (1‐h or 2‐h) and a combination of fasting and postglucose load abnormalities with future type 2 diabetes

Isolated FPG, postglucose load (1‐h or 2‐h) PG, and a combination of FPG and postglucose load PG abnormalities were seen in 29.4%, 27.7%, and 42.9% participants, respectively (Table 2]. The corresponding incidence rates for diabetes were 3.8, 5.9, and 14.3/100 women years (Table 3). The adjusted hazard ratio for the combined abnormalities was 2.61 (95% CI, 1.70–4.00; *p* < .001), compared to isolated abnormality of FPG (Table 4).

### 3.4 Association between the number of values deranged and future risk for type 2 diabetes

The incidence rates for participants with one, two, and three abnormal values were 4.3, 8.3, and 19.0/100 women‐years, respectively. The adjusted hazard ratio for two and three abnormal values compared to one abnormal value was 1.73 (95% CI, 1.18–2.54; *p* = .005) and 3.56 (95% CI, 2.46–5.16; *p* < .001), respectively.

## 4 DISCUSSION

**FIGURE 2:**
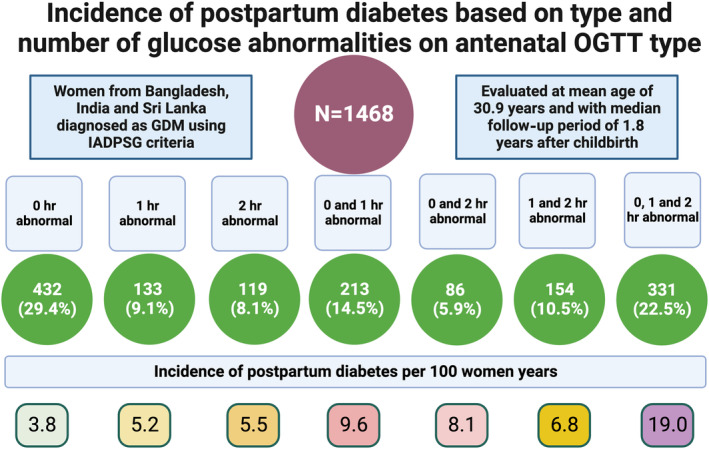
Key findings of this study. GDM, gestational diabetes; IADPSG, International Association of the Diabetes and Pregnancy Study Groups; OGTT, oral glucose tolerance test.

We evaluated 1468 women at a median of 1.8 (1.4–2.4) years after childbirth from the index pregnancy with GDM. We found diabetes in 213 (14.5%) women with an incidence rate of 8.7 (7.6–10.0)/100 women‐years. The incidence rate varied significantly from 3.8/100 women‐years in those with isolated FPG abnormality (constituting 29.4% of the total women) to 19.0/100 women‐years in those with all three abnormal values in OGTT (constituting 22.5% of the total women). The incidence rate was 8.3/100 women‐years when two values were abnormal compared to 4.3/100 women‐years when only one was abnormal. The results suggest a marked gradation in the risk of future diabetes, depending upon the number of values or type of abnormalities on an antenatal OGTT conducted for diagnosing GDM. We have summarized the main findings in Figure 2.

A meta‐analysis of 17 studies (with publication dates up to February 2020) to assess the association between antepartum OGTT and the future risk of diabetes in women with GDM, found that the odds for future diabetes were 3.62 and 3.96 when fasting or 2‐h OGTT values respectively were elevated.^7^ Though informative, the meta‐analysis is less relevant in the current scenario, as the studies were all done before 2010, when women with overt diabetes were included with GDM. None of the past studies used IADPSG criteria, which are currently broadly adopted. There was also no representation from South Asia. Our study overcomes some limitations in this meta‐analysis and previous research and also has a participant sample (*n* = 1468) greater than in the largest study (*n* = 1263) in the meta‐analysis.

A 2023 study of 177 241 women in Israel reported an association of the incidence of diabetes for women with prior GDM who were diagnosed based on a two‐step strategy, with OGTT conducted by Carpenter and Coustan criteria.^17^ The diabetes incidence was 3.4‐fold for women with abnormal glucose challenge test and normal OGTT, and 9.1‐fold higher in women with an abnormal value on OGTT at 10.8 years of median follow‐up after childbirth. Women with an abnormal challenge and an isolated abnormal FPG had 11.8‐fold higher risk for future diabetes. The risk of future diabetes varied from 11‐ to 61‐fold higher depending upon the number of values deranged. The rates reported in this study are higher, possibly due to the two‐stage testing, longer follow‐up, and values extending to four in the Carpenter and Coustan criteria compared to three in the IADPSG criteria.

Another recent study explored the association between antenatal OGTT and the future risk of diabetes for women diagnosed with GDM using IADPSG criteria in 20 513 women in Ontario, Canada.^8^ Based on median follow‐up of 4.4 years, the overall incidence rates were 1.4 and 0.2 per 100 women‐years in women with and without GDM, respectively. The incidence rates for future diabetes based on one, two, or three abnormal values were 0.7, 1.6, and 4.0/100 women‐years respectively. The corresponding rates in our study were 4.3, 8.3, and 19.0/100 women years. The risk for future diabetes in women with three, compared to one abnormal OGTT value, was 5.7‐fold higher in the study in Canada and 4.4‐fold higher in our study in South Asia. The incidence rates for future diabetes based on 0 h, 1‐h, and 2‐h values in Canada were 2.5, 1.7, and 1.7/100 women years. The corresponding figures were 9.8, 11.9, and 12.4/100 women years in our study. Interestingly, the diabetes rates were lower with FPG abnormality in our study compared to those in Canada. Even in the antepartum period, the studies in other ethnicities suggest that women having abnormal FPG compared to 1‐h and/or 2‐h abnormalities have unfavorable metabolic profiles in early pregnancy and higher rates of pregnancy complications and requirement of insulin in pregnancy.^18^, ^19^, ^20^ There have been limited data on postpartum diabetes. It will be crucial to explore ethnic differences in postpartum glycemia based on antenatal OGTT values and mechanistic insights in future studies. A study in Singapore with 942 women and a follow‐up of 6–12 weeks postpartum found a relative risk of 44.5 for future diabetes, when all three OGTT values were abnormal, compared to isolated FPG abnormality. In contrast, we found a hazard ratio of 3.60 (2.30–5.64) for a similar comparison. The results suggest that the degree of association between antenatal OGTT phenotype and future diabetes vary significantly by country and ethnicity. The variation in findings could also be due to differences in follow‐up periods, demographic factors, and the relative contribution of different pathophysiological defects in the pathogenesis of diabetes.

It is logical to assume that the risk of future diabetes should increase as the number of abnormal values on antenatal OGTT increases. However, from a public health perspective, precise information helps plan targeted interventions for diabetes prevention. In the LIVING study, we found no benefit of a low‐intensity lifestyle intervention for behavior modification,^13^ and we propose that either a high‐intensity intervention or pharmacotherapy, such as metformin, should be evaluated in this high‐risk South Asian population. However, the reality is that the health care system does not have the resources to deliver high‐intensity lifestyle interventions, nor will all women be willing to take pharmacotherapy to prevent diabetes. Therefore, there is a need to identify from a broader pool of women with GDM, diagnosed based on pregnancy risks, those who are at highest risk for future diabetes. For instance, if we define women with annual incidence rates of >10/100 women years at high risk for future diabetes, this includes women with at least two abnormal values (including an abnormal FPG value) (42.9%). Similarly, if an annual incidence rate of <5/100 women years is classified as low risk, individuals with isolated FPG abnormality (29.2%) will fall in this category. This simple information can inform broad risk groupings and enable targeted interventions. This classification into low‐, intermediate‐ (5–10/100 women‐years), and high‐risk categories can help select women for different interventions. Such an approach can also inform follow‐up monitoring strategies required for women with different levels of risk.

Strengths and limitations: This study reports data on the future risk of diabetes based on the type of antenatal OGTT abnormality and is the second largest study, the only one in South Asia, and applies the IADPSG criteria. Our findings inform the planning of future trials and monitoring in this field, especially in South Asia. The study has some limitations: data are predominantly from urban centers and cannot be generalized to the whole South Asian population. Follow‐up is relatively short, and the degree of association may change with longitudinal follow‐up, with an anticipated increase in event rates.

Conclusion: This is the first such study from South Asia using IADPSG criteria for the diagnosis of diabetes. Risk of future diabetes was high in South Asian women and varied significantly depending upon the type and number of abnormal values on antenatal OGTT. The most prevalent abnormality was an elevated FPG in 72.3% of women, with 29.4% with one elevated value having a high FPG. The incidence rate of diabetes varied by OGTT abnormality and combinations and was 19.0 /100 women years with all elevated values compared to 3.8 /100 women years with isolated FPG abnormality. Overall, women with only one abnormal value had relatively low risk (incidence <5/100 women‐years) of postpartum diabetes. Our findings could guide targeted approaches for ongoing OGTT monitoring and inform future interventions, based on simple available measures from antenatal OGTTs, especially for the urban South Asian population, where the future risk of diabetes is relatively high, compared to other ethnicities.

## AUTHOR CONTRIBUTIONS

Nikhil Tandon and Anushka Patel conceived the study and obtained funding. Yashdeep Gupta wrote the first draft. Joseph Alvin Santos did the statistical analysis. All other authors contributed to concept, design, or operational aspects the study and contributed to revisions of the draft. Nikhil Tandon and Anushka Patel had full access to all of the data in the study and take responsibility for the integrity of the data and accuracy of the data analysis. All members of the LIVING Collaborative Group are listed here: Md Muniruzzaman Siddiqui, Ishrat Jahan, Mohammad Hussain Chowdhury, Md Faruque Pathan, Bishwajit Bhowmik, Prema Varthakavi, Nikhil Bhagwat, Vaibhavi Mungekar, Rekha Fernandes, Vrinda Pednekar, Nalini Shah, Tushar Bandgar, Swati Jhadav, Arti Utekar, Urjita Ramchandra Sarnobat, Neelam Jaguste, Arti More, Kedar Narvenkar Narvenkar, Guruprasad Padnekar, Ajit Arvind Nagarsenkar, Sachina Vithu Satarkar, Praciya Shyam Goankar, Sneha Chari, Retakshi Ghadi, Nupur Phadte, Mabel Anne Alvares, Mrunali Gaude, Shiwani Dadwal, Sailee Prabhu, Sanjay Bhadada, Neelam Aggarwal, Chandana Datta, Seema Dahiya, Deepak Khandelwal, Soniya Chahal, Renu Mann, Rajiv Singla, Monika Bhatia, Geetu Gupta, Bharti Kharal, Sadishkumar Kamalanathan, Rajan Palui, Jaya Prakash Sahoo, Papa Dasari, Niya Narayan, Varun Suryadevara, Kavi Priya, R Priya, V Mohan, Uma Ram, Guha Pradeepa, Rajasree Gopinath, R Krishnaveni, U Ashwini, E Chandralekha, P Nandhini, Mala Dharmalingam, Chitra Selvan, Pramila Kalra, Mamta Sanjeeva, Dev Sreenivasa, Sowmya G S, Nihal Thomas, Sahana Shetty, Felix Jebasingh, Riddhi Dasgupta, Jiji Mathew, Kavitha Sankar, Jansi Vimala Rani, Nithya Devanithi, Flory Christina, Shirley Newton, Anisha Gala, S Tarakeswari, Vidyavati Patil, M Bhavana Reddy, K Vijayalakshmi, G Vasantha Rani, Sunil Fernando, Carmaline Motha, Sanjeeda Baduge, Sachini Rangana Withanage Withanage, Dilumi Jayawickrama, Saumya Hapuarachchi, Sripali Amarasinghe, Athula Kaluarachchi, Mnm Rishad, Sachini Ranasinghe, Madara Jayanetti, Aaisha Azam, Ravija Ramasinghe, H V L Rangika, Rukshan Fernandopulle, Madura Jayawardena, Sheran Siyambalapitiya, Gayan Liyanage, Piyumalie Hettiarachchi, Dimuthu Kaluarachchi, Uthpala Chandradeva, Kalawila Withanage, Rangana Amarasinghe, Deepa Nandani, Jeyakumar Sabaretnam, Malaka Amarasena, M D Radhika Sriyakanthi, Kanchana Peiris, Thiyagarajah Kadotgajan, Milinda Gamlath, Asha Weerasinghe, A H Samanthi, Anu Kaushik, Neha Sethi, Ishita Agarwal, Vandana Garg, Kanika Chopra, Divya Soni, Purnima Rao Jevaji, Pavitra Madhira, Thanushanthan Jeevaraja, Shehan Gnanapragasam, Shabnam Sheuly, Nazia Ferdowsh, Tarana Mustari, Shahnaz Parvin Munni, Azmira Khatun, Marzia Sultana, Rifat Hasan Shammi, Sabrina Ahmed, Nantu Chakma, Helen Monaghan, Sindhu Prasad, Amrutha Nagarajaiah, Prakash Velappan, Koushik Gade, Swathi Pagadala, Prithvishree B Radhakrishna, Ambika Yoganathan, Sumathi Senthil, Ravikumar Tummapudi, Ullas Arabhavi, Catherine Lombard, Dewan Alam. All named authors meet the International Committee of Medical Journal Editors criteria for authorship for this article, take responsibility for the integrity of the protocol as a whole, and have given their approval for this version to be published.

## FUNDING INFORMATION

This study was funded by Global Alliance for Chronic Disease grants from the Indian Council of Medical Research (No. 58/1/1/GACD/NCD‐II) and National Health and Medical Research Council of Australia (1093171). Additional funding was received from USV Pharmaceuticals Ltd and Lupin Pharmaceuticals Ltd for substudies (data not reported here). The funders had no role in the design and conduct of the study; collection, management, analysis, and interpretation of the data; preparation, review, or approval of the manuscript; and decision to submit the manuscript for publication.

## DISCLOSURES

Dr Zoungas reported participation in expert committees or educational meetings outside the submitted work on behalf of Monash University, which received payment from Boehringer‐Ingelheim, Eli Lilly, Sanofi, AstraZeneca, Novo Nordisk, and Merck Sharp and Dohme. The authors have no other disclosures to declare. Anushka Patel is an Editorial Board member of the *Journal of Diabetes* and a coauthor of this article. To minimize bias, she was excluded from all editorial decision‐making related to the acceptance of this article for publication.

## PATIENT CONSENT STATEMENT

We enrolled participants after written informed consent from each participating individual.

## Data Availability

Data, protocols, and all documentation pertaining to the analyses presented here will be made available to academic and other researchers after approval of a Data Access Request. A request form can be obtained by emailing dsc@georgeinstitute.org.
